# Leadership in nursing: why should we discuss it?

**DOI:** 10.26633/RPSP.2019.46

**Published:** 2019-05-03

**Authors:** Silvia Helena de Bortoli Cassiani, Maria Neyrian de Fatima Fernandes, Kimberly Lecorps, Fernando Antonio Menezes da Silva

**Affiliations:** 1 Health Systems and Services Department Health Systems and Services Department Pan American Health Organization/World Health Organization WashingtonD.C. United States of America Pan American Health Organization/World Health Organization, Health Systems and Services Department Washington, D.C., United States of America; 2 Nursing School Nursing School Universidade Federal do Maranhão Imperatriz, Maranhão Brazil Universidade Federal do Maranhão, Nursing School Imperatriz, Maranhão, Brazil; 3 Pan American Health Organization/World Health Organization Pan American Health Organization/World Health Organization WashingtonD.C. United States of America Pan American Health Organization/World Health Organization Washington, D.C., United States of America; 4 Health Systems and Services Department Health Systems and Services Department Pan American Health Organization/World Health Organization WashingtonD.C. United States of America Pan American Health Organization/World Health Organization, Health Systems and Services Department Washington, D.C., United States of America

Dear Editor:

The Nursing Now campaign, which will end in 2020, involves a collaborative partnership of the United Kingdom’s Burdett Trust for Nursing, the World Health Organization (WHO), and the International Council of Nurses. The campaign aims to improve health around the world by raising the profile and status of nurses globally, influencing policymakers, and supporting nurses to lead, learn, and build a worldwide movement. The campaign advocates for more nurses in positions of leadership and for enhancing their influence on national and international policies to address the current social issues that affect health care systems. Nurses have direct interaction and build trust with the general public. They are involved in all levels of health care delivery and can serve as instrumental leaders in transforming the health care system.

Today’s society and education systems offer a unique opportunity for nurses to develop leadership skills. However, existing barriers prevent nurses from assuming more leadership positions. Issues in the distribution of nurses, women’s empowerment, and sociocultural factors such as gender have affected the professional progression of nurses.

In the Region of the Americas, a shortage of nurses impacts access to qualified health personnel. In many countries, there are an unequal distribution and a low ratio of nurses to support health services. In addition, a scarcity of educators constrains education and training. Nevertheless, to address the shortage, strategies often call for producing more nurses to meet the demand, but those new professionals are frequently not adequately prepared to function and lead in an increasingly complex, strained workplace (1).

Given the low ratio of nurses, the emphasis remains on addressing immediate priorities. In many countries, nursing education and training now focus more on a disease-based approach than on health promotion and prevention (1). The WHO has called for a shift to a people-centered health model (2). Nurse leaders will be responsible for facilitating this transformation and preparing the future work force. Effective nurse leadership will ensure judicious strategic planning, implementation, and evaluation in increasing the number of nurses and preparing them to meet the population’s needs.

Another concern is that nurses are not empowered to assume leadership positions. It is essential to bolster leadership skills in nursing education, at both the undergraduate and graduate level. Nursing degree programs and continuing education should strengthen nurses’ leadership potential. It is crucial to include both practical skills and health policy development in the academic curriculum. A systemic vision instilled during professional training and clinical practice can empower nurses.

Sociocultural factors related to gender are also an important concern, since most nurses in the Region of the Americas are women. Even though women have attributes considered effective for leadership, such as sensitivity and empathy, there are lingering gender gaps in leadership positions. Women face more structural barriers and unequal expectations than do men. Women are also underrepresented in senior positions in both health systems and the political realm in most countries of the world (3-5).

The Region of the Americas needs more nurses working in the formulation, implementation, and execution of public health policies, at both the local and national level of government. The demands and complexity of health systems require professionals capable of mobilizing cognitive and material resources to propose and formulate policies. There must also be professionals capable of working interprofessionally and making fundamental decisions that will improve working conditions, expand universal access and coverage, and promote societal well-being. Nurses can respond to these needs if they are given new opportunities and are allowed to work to their full potential.

To empower, support, and discuss nurse leadership in the Region of the Americas, the Pan American Health Organization (PAHO) has developed the Strategic Direction for Nursing in the Region of the Americas, which aligns with the WHO Global Strategic Directions for Strengthening Nursing and Midwifery 2016-2020 (6) and also supports the Nursing Now campaign (7). (After May 8, the PAHO Strategic Direction publication will be available at: https://www.paho.org/hq/index.php?option=com_content&view=article&id=11186:publications-nursing&Itemid=41557&lang=en.)

The Strategic Direction for Nursing in the Region of the Americas presents three lines of action. The first proposes strengthening and consolidating the leadership and strategic management of nursing in the context of health systems and in the formulation and monitoring of policies. The second is related to nurses’ working conditions and their skills for expanding access and coverage with equity and quality. The last line of action aims at strengthening the quality of and educational preparation for nursing, in order to better respond to the needs of health systems as they work toward universal access and health coverage and achieving the Sustainable Development Goals (7).

The Strategic Direction for Nursing in the Region of the Americas was formulated by taking into account the possibilities for nurses to use their leadership skills and effectively contribute to the transformation of health care systems. The current health landscape needs dynamic, empowered professionals to occupy key positions. However, these opportunities must be created. It is necessary to bring nurses to the discussion table, including giving them a voice, professional recognition, a positive working environment, compatible salaries, and opportunities for professional development.

Establishing conditions that encourage leadership and create opportunities for nurses to advance professionally will ensure substantial benefits for society.

## Disclaimer.

Authors hold sole responsibility for the views expressed in the manuscript, which may not necessarily reflect the opinion or policy of the *RPSP/PAJPH* and/or PAHO.

**Silvia Helena de Bortoli Cassiani**

Pan American Health Organization/World Health Organization, Health Systems and Services Department

Washington, D.C., United States of America

cassianis@paho.org

**Maria Neyrian de Fatima Fernandes**

Universidade Federal do Maranhão, Nursing School

Imperatriz, Maranhão, Brazil

**Kimberly Lecorps**

Pan American Health Organization/World Health Organization

Washington, D.C., United States of America

**Fernando Antonio Menezes da Silva**

Pan American Health Organization/World Health Organization, Health Systems and Services Department

Washington, D.C., United States of America
